# Does Maturity Affect Cephalic Perfusion and T/QRS Ratio during Prolonged Umbilical Cord Occlusion in Fetal Sheep?

**DOI:** 10.1155/2014/314159

**Published:** 2014-02-16

**Authors:** Guido Wassink, Robert Galinsky, Paul P. Drury, Eleanor R. Gunn, Laura Bennet, Alistair J. Gunn

**Affiliations:** Fetal Physiology and Neuroscience Group, Department of Physiology, University of Auckland, Auckland 1023, New Zealand

## Abstract

T/QRS ratio monitoring is used to help identify fetal asphyxia. However, immature animals have greater capacity to maintain blood pressure during severe asphyxia, raising the possibility that they may show an attenuated T/QRS increase during asphyxia. Chronically instrumented fetal sheep at 0.6 of gestation (0.6 GA; *n* = 12), 0.7 GA (*n* = 12), and 0.8 GA (*n* = 8) underwent complete umbilical cord occlusion for 30 min, 25 min, or 15 min, respectively. Cord occlusion was associated with progressive metabolic acidosis and initial hypertension followed by severe hypotension, with a more rapid fall in mean arterial blood pressure (MAP) and carotid blood flow (CaBF) with advancing gestation. T/QRS ratio rose after occlusion more rapidly at 0.8 GA than in immature fetuses, to a similar final peak at all ages, followed by a progressive fall that was slower at 0.8 GA than in the immature fetuses. The increase in T/QRS ratio correlated with initial hypertension at 0.8 GA (*P* < 0.05, *R* ^2^ = 0.38), and conversely, its fall correlated closely with falling MAP in all gestational groups (*P* < 0.01, *R* ^2^ = 0.67). In conclusion, elevation of the T/QRS ratio is an index of onset of severe asphyxia in the last third of gestation, but not of fetal compromise.

## 1. Introduction

Brain injury from perinatal asphyxia remains a significant cause of mortality and long-term morbidity in newborn infants [1, 2]. Experimentally, the development of hypoxic-ischemic neural injury is broadly associated with the degree and duration of exposure to systemic hypotension and cerebral hypoperfusion [3–6]. Previous evidence suggests that cerebrovascular autoregulation is relatively immature in fetal sheep at 0.6 and 0.7 of gestational age (GA) [7, 8], with cerebral blood flow falling in parallel with development of hypotension [9]. Although cerebral autoregulatory capacity increases with maturation [10], even in 118–122 day old fetal lambs resting blood pressure is very close to the lower limits of the autoregulatory range [11]. Further, isolated hypercarbia can impair regional cerebral autoregulation in healthy, normotensive newborn piglets [12]. Conversely, we have previously shown that the immature fetus at 0.6 and 0.7 GA can maintain its arterial blood pressure for much longer during prolonged asphyxia than at 0.8 GA [13], suggesting later impairment of cerebral perfusion in immature fetuses. Collectively, these findings indicate a critical maturation dependent neural vulnerability of the developing fetus to hypotension during asphyxia, but the exact quantitative relationship between hypotension and cerebral perfusion from 0.6 to 0.8 GA remains unclear.

**Figure 1:**
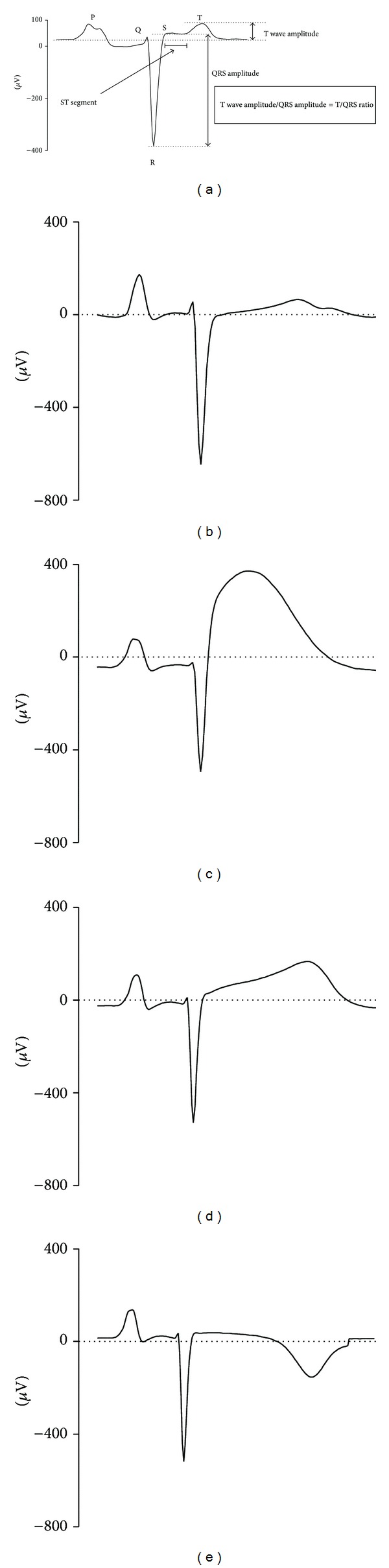
Examples of the ECG complex taken from a 0.7 gestation fetus. (a) A representative fetal ECG complex with identification of the ST segment, T wave amplitude, and QRS amplitude. The T/QRS ratio is calculated for each complex by division of the T wave amplitude by the QRS amplitude, thus correcting for changes in signal gain from electrode placement and fetal movement. (b) The ECG complex during the baseline period. (c) Characteristic ST segment elevation early during the occlusion when the fetus is compensating well. (d) Gradual reduction in ST segment elevation concordant with a fall in T/QRS ratio as the fetus starts to compromise from prolonged asphyxia. (e) Progressive T wave inversion immediately after prolonged severe asphyxia.

Bedside clinical surveillance with electronic fetal monitoring would ideally be able to readily identify those infants that are at risk of developing hypotension. ST segment augmentation of the fetal electrocardiogram, measured relative to the QRS complex to correct for changes in signal gain (the T/QRS ratio, as illustrated in Figure 1), is a well-established marker for the presence of fetal asphyxia in both clinical and experimental studies [14–17]. Increased T/QRS is believed to reflect anaerobic cardiac metabolism with consumption of myocardial glycogen reserves [18]. For example, the rate of myocardial glycogenolysis during graded hypoxia, with depletion of glycogen and ATP, was directly associated with the degree and rate of ST segment elevation in acutely exteriorized fetal lambs [18, 19].

These studies typically involved relatively moderate asphyxia. During severe hypoxia in full term fetal lambs leading to a progressive fall in arterial pressure, brief elevation of the ST segment was followed by a fall in amplitude that correlated with the severity of metabolic acidosis [19]. This is consistent with studies in fetal sheep at 91–94 and 123–129 days of gestation that demonstrated a brisk transient rise in the T/QRS ratio, associated with initial hypertension during umbilical cord occlusion, and a gradual reduction in T/QRS ratio as fetal hypotension and metabolic acidosis progressively worsened [17, 20].

The capacity of the immature fetus to maintain systemic pressure more effectively during asphyxia has been attributed to factors such as greater anaerobic capacity, lower basal metabolic activity, and greater cardiac glycogen stores, which are known to be the highest at midgestation [21–23]. Notably, circulating catecholamines, which augment cardiac contractility and peripheral vasoconstriction during occlusion [24], increase substantially towards term gestation. There is evidence that this *β*-adrenoreceptor stimulation contributes to the increase in T/QRS ratio [25].

Taken together, these findings suggest the hypothesis that maturational differences in blood pressure response during prolonged asphyxia may be reflected in the T/QRS ratio. The T/QRS ratio alone, however, may not reliably identify the onset of hypotension during severe asphyxia in fetal sheep [17, 26, 27]. Alternatively, changes in the shape of the ECG waveform such as biphasic ST waveforms and inverted T waves have been suggested to be more reliable markers of cardiac metabolic compromise [26]. For example, ST segment depression with inverted T waves was observed in growth retarded fetal guinea pigs during maternally induced hypoxia, whereas their normally grown littermates showed ST elevation [28]. Similarly, the appearance of biphasic ST waveforms and inverted T waves was associated with the onset of severe decompensation during repeated brief umbilical cord occlusions in near-term fetal sheep [26].

Thus, in the present study we sought to examine the quantitative relationship between arterial blood pressure and cephalic blood flow during prolonged umbilical cord occlusion in fetal sheep at 0.6, 0.7, and 0.8 GA. In addition, we investigated whether better maintenance of systemic blood pressure in immature fetuses was reflected by differences in the T/QRS ratio or changes in waveform shape. These ages are broadly equivalent to the neural maturation of the human fetus at 26–28, 28–32 and 40 weeks of gestation, respectively [29].

## 2. Materials and Methods

### 2.1. Surgical Preparation and Postoperative Care

All procedures were approved by the Animal Ethics Committee of The University of Auckland, New Zealand. Under general anesthesia, Romney/Suffolk singleton fetal sheep were instrumented at 86–89 days of gestation (0.6 GA; term = 147 days), 98-99 days (0.7 GA), or 117–122 days (0.8 GA) using sterile techniques as previously described [13]. We have previously reported changes in fetal heart rate (FHR), mean arterial pressure (MAP), femoral blood flow (FBF), femoral vascular conductance (FVC), and blood gas parameters in a subset of these animals [13].

Food, but not water was withdrawn 18 h before surgery. Ewes were given 5 mL of Streptopen (procaine penicillin (250,000 IU/mL) and dihydrostreptomycin (250 mg/mL), Pitman-Moore, Wellington, New Zealand) intramuscularly for prophylaxis 30 min prior to the start of surgery. Anesthesia was induced by intravenous (i.v.) injection of Alfaxan (Alphaxalone, 3 mg/kg, Schering-Plough Animal Health Ltd., Wellington, New Zealand) and general anesthesia maintained using 2-3% isoflurane in O_2_. The depth of maternal anesthesia, heart rate, and respiration were monitored constantly by trained anesthetic staff, and the ewes received a constant infusion of isotonic saline to maintain fluid balance.

Following a maternal midline abdominal incision and exteriorization of the fetus, polyvinyl catheters were placed in the left and right brachial artery and vein to measure arterial blood pressure and to obtain fetal arterial blood samples. An amniotic catheter was secured to the fetal shoulder. An ultrasound blood flow probe (probe type 3S, Transonic Systems Inc., Ithaca, NY, USA) was placed around the left carotid artery to measure carotid blood flow (CaBF) as an index of global cephalic blood flow [30, 31]. A stainless steel electrode (Cooner Wire Co., Chatsworth, CA, USA) was placed across the fetal chest to measure the fetal electrocardiogram (ECG), and an inflatable silicone occluder was placed around the umbilical cord of all fetuses (In Vivo Metric, Healdsburg, CA, USA). All fetal leads were exteriorized through the maternal flank and a maternal long saphenous vein was catheterized to provide access for postoperative care and euthanasia. Antibiotics (80 mg Gentamicin, Pharmacia and Upjohn, Rydalmere, NSW, Australia) were administered into the amniotic sac prior to closure of the uterus. Amniotic fluid lost during surgery was replaced with normal saline warmed to 37°C. The maternal wall was repaired and the skin incision infiltrated with a local analgesic, 10 mL 0.5% bupivacaine plus adrenaline (AstraZeneca Ltd., Auckland, New Zealand). Postoperatively, ewes were housed together in individual metabolic cages with access to water and concentrate feed (Country Harvest Stockfeed, Cambridge, New Zealand) *ad libitum *in a temperature-controlled housing facility (16 ± 1°C, humidity 50 ± 10%) on a 12 h light-dark cycle. Antibiotics were given daily for five days after surgery and administered i.v. to the ewe (600 mg Benzylpenicillin Sodium, Novartis Ltd, Auckland, New Zealand, 80 mg Gentamicin). Fetal catheters were maintained patent by continuous infusion of heparinized saline (10 U/mL at 0.2 mL/h at 0.6 GA and 20 U/mL at 0.2 mL/h at 0.7 and 0.8 GA, resp.) and the maternal catheter maintained by daily flushing.

### 2.2. Data Acquisition

Fetal MAP (Novatrans II, MX860, Medex Inc., Hilliard, OH, USA), corrected for maternal movement by subtraction of amniotic pressure, CaBF, and ECG, were recorded continuously throughout the experiment. The blood pressure signals were collected at 64 Hz and low pass filtered at 30 Hz. The fetal ECG was analog filtered between 0.05 and 80 Hz and digitized at 512 Hz. All experimental data were collected using customized acquisition software (LabVIEW for Windows, National Instruments Ltd., Austin, Tx, USA) for offline analysis.

### 2.3. Experimental Design

Experiments were conducted 4 to 5 days after surgery, at 91 ± 1 days of gestation (0.6 GA, *n* = 12), 104 ± 1 days (0.7 GA, *n* = 12), or 125 ± 1 days (0.8 GA, *n* = 8). Fetal asphyxia was induced by rapid inflation of the umbilical occluder with sterile saline of a predefined volume known to totally compress the umbilical cord, as determined in pilot experiments with an ultrasound blood flow probe placed around an umbilical vein [32]. The duration of occlusion was chosen to represent an acute, near-terminal insult for each age, and established at 30 min at 0.6 GA [32, 33], 25 min at 0.7 GA [34, 35], and 15 min at 0.8 GA [17, 36]. Adrenalin (0.1 mL/kg estimated fetal weight, 1 : 10000 epinephrine, Health Support Ltd., Auckland, New Zealand) was given i.v. to the fetus if bradycardia persisted for more than 60 seconds after release of occlusion.

Fetal blood composition analysis (Ciba-Corning diagnostics 845 blood gas analyzer and co-oximeter, MA, USA) and measurements of glucose and lactate levels (YSI model 2300, Yellow Springs, OH, USA) were performed 60 min prior to occlusion (baseline), during the occlusion (0.6 GA at 5 and 25 min, 0.7 GA at 5 and 20 min, and 0.8 GA at 2 and 12 min), and 60 min after the occlusion (recovery). After the experiment, ewes and fetuses were euthanized by an i.v. overdose of pentobarbitone sodium to the ewe (9 g, Pentobarb 300; Chemstock International, Christchurch, New Zealand). Fetuses were then removed by hysterectomy and weighed.

### 2.4. Data Analysis and Statistical Procedures

Offline analysis of the physiological data was performed using custom analysis programs (LabVIEW for Windows). 5 min (baseline period), 5 sec, and 1 min averages of T/QRS ratio, FHR, MAP, and CaBF were calculated for each fetus, respectively. The 5 sec averaged data were used to assess detailed changes in fetal hemodynamics during the first 10 minutes of umbilical cord occlusion while the one-minute data were used to assess the remaining occlusion time points. The baseline period was taken as the mean of one hour before occlusion. Because of the large differences in absolute values for the T/QRS ratio, FHR, MAP, and CaBF variables with advancing gestation, parameters were converted to percent changes from baseline, with exception of T/QRS ratio which was normalized to baseline.

The electrocardiogram waveform (derived from the fetal ECG) was averaged with respect to the S wave over 5 sec intervals. For each averaged waveform, the ratio between the T wave height (measured from the level of the PQ interval) and the total QRS amplitude was calculated (T/QRS ratio, Figure 1). Visual assessment of these waveforms was performed by a blinded observer to confirm correct software identification of the T wave and to identify the presence of any ST waveform anomalies, such as biphasic ST waveforms or negative T waves. Two fetuses in the 0.6 and 0.7 gestation group were not included in these analyses because of poor signal quality.

The effects of gestation and occlusion on physiological and blood composition data were assessed by ANOVA (SPSS v16, SPSS Inc., Chicago, IL, USA). Changes over time were treated as a repeated measure. Where statistical significance was detected, post hoc comparisons were performed with univariate analysis and Fisher's Least Significant Difference test or Dunnett's test when compared to baseline. The within-subjects relationship between MAP, CaBF, and T/QRS ratio for selected occlusion periods (1 min data) was determined by regression analysis using the methodology of Bland and Altman [37]. Statistical significance was accepted when *P* < 0.05. Data are mean ± SEM.

## 3. Results

Baseline T/QRS ratio, FHR, MAP, and CaBF are shown in Table 1. Briefly, baseline T/QRS ratio and FHR was significantly lower at 0.8 GA, while MAP and CaBF increased significantly with advancing gestation. Fetal weight (0.6 GA; 1283 ± 155 g, 0.7 GA; 1623 ± 126 g, 0.8 GA; 3329 ± 170 g) was the greatest at 0.8 GA (*P* < 0.05 versus 0.6 GA and 0.7 GA). Two fetuses in the 0.6 GA group and one fetus in the 0.7 GA group had their occlusions released early (at 23 min, 25 min, and 20 min, resp.) following acute heart block. Four of 8 fetuses in the 0.8 GA group died from terminal cardiac failure within 4 hours after release of the occlusion, and two additional fetuses died within 24 hours. All fetuses from the 0.6 and 0.7 GA groups survived after occlusion.

### 3.1. Blood Composition Analysis

Before the experiment, all fetuses had normal blood gases, pH, lactate, and glucose values for their age (Table 2) according to the standards of our laboratory. Complete prolonged occlusion of the umbilical cord resulted in profound hypoxia and development of severe mixed metabolic and respiratory acidosis in each fetus, with more severe values in the younger fetuses than at term, reflecting the longer durations of occlusion. Plasma lactate levels during the immediate recovery period were the highest at 0.8 GA.

### 3.2. Fetal Heart Rate, Mean Arterial Pressure, and CaBF

**Figure 2:**
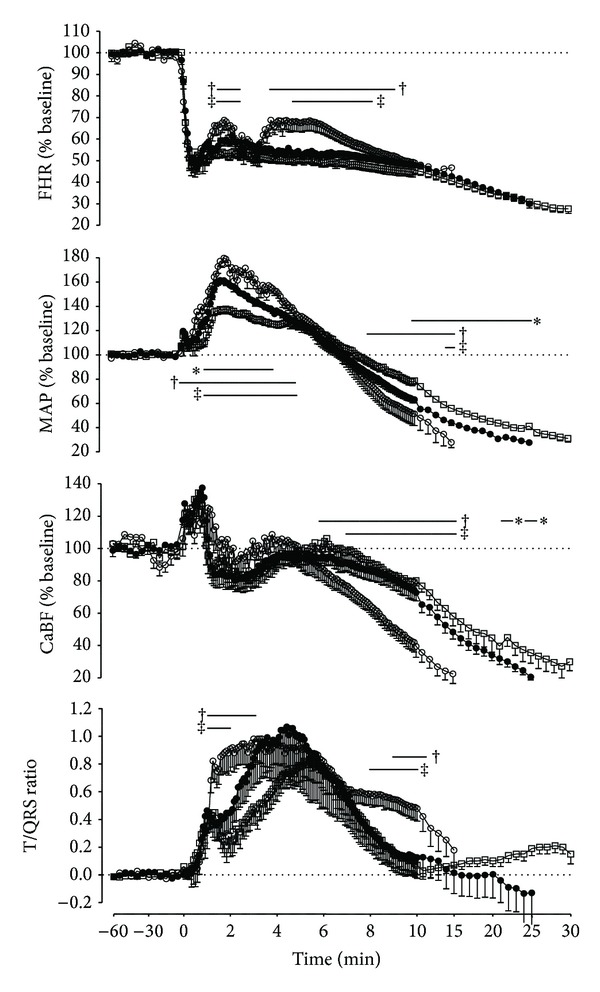
Changes in fetal heart rate (FHR, % baseline), mean arterial pressure (MAP, % baseline), carotid blood flow (CaBF, % baseline), and T/QRS ratio in 0.6 gestation (0.6 GA; *▫*), 0.7 gestation (0.7 GA; ∙), and 0.8 gestation (0.8 GA; ∘) fetuses during prolonged umbilical cord occlusion. Data are 5 min averages before occlusion, 5 sec averages during the first 10 minutes of occlusion, and 1 min averages for all other occlusion time points, expressed as percentage of baseline. The period of umbilical cord occlusion for each group begins at 0 min and ends at 15 min (0.8 GA), 25 min (0.7 GA), and 30 min (0.6 GA), respectively. Data are mean ± SEM; between-group comparisons by repeated measures ANOVA and LSD test. *P < 0.05; 0.6 GA versus 0.7 GA, ^†^ *P* < 0.05; 0.6 GA versus 0.8 GA, ^‡^ *P* < 0.05; 0.7 GA versus 0.8 GA.

Umbilical cord occlusion was associated with a rapid initial fall in FHR from baseline in all gestational groups by 61.2 ± 1.5% within the first minute; the relative magnitude of this fall was not different between groups (N.S.). With the exception of a transient increase in FHR at 2 min and again between 4 and 9 min of occlusion at 0.8 GA (*P* < 0.05 versus 0.6 GA and 0.7 GA, Figure 2), FHR showed a consistent and linear fall throughout the occlusion in all groups. The FHR nadir at the end of occlusion (relative nadir, 0.6 GA: 27.6 ± 1.8%, 0.7 GA: 30.7 ± 1.6%, 0.8 GA: 46.7 ± 3.8%, and absolute nadir, 0.6 GA: 52.1 ± 3.0 bpm, 0.7 GA: 58.5 ± 3.5 bpm, 0.8 GA: 80.0 ± 6.6 bpm) was significantly lower in the younger groups (*P* < 0.01; 0.6 GA and 0.7 GA versus 0.8 GA, Figure 2). The immature groups (0.6 GA and 0.7 GA) showed immediate recovery of FHR and MAP after release of the occlusion, whereas 6 of 8 0.8 GA fetuses required adrenalin for cardiac resuscitation.

Occlusion was associated with a rapid rise in MAP, to a peak after 1.9 ± 0.1 min (N.S. between groups). The magnitude of hypertension increased proportionally towards near-term gestation (relative increase in MAP: 0.6 GA: 144.4 ± 2.8%; 0.7 GA: 165.1 ± 2.7%; 0.8 GA: 189.5 ± 1.5%; absolute increase in MAP: 0.6 GA: 51.7 ± 1.3 mmHg; 0.7 GA: 61.0 ± 1.3 mmHg; 0.8 GA: 82.6 ± 1.2 mmHg; *P* < 0.01, 0.6 GA versus 0.7 GA versus 0.8 GA, Figure 2). MAP then declined progressively over time, falling below baseline from 7.7 ± 0.3 min onwards (N.S. between groups). Ultimately, severe systemic hypotension developed in all gestational groups. The overall rate of fall in MAP during the occlusion increased substantially with advancing gestational age, with a proportionately lower MAP at 0.8 GA than 0.6 GA from 8 min onwards (*P* < 0.05) and than 0.7 GA at 15 min of occlusion (*P* < 0.05). Consequently, MAP was also significantly lower at 0.7 GA from 10 min of occlusion onwards (*P* < 0.05 versus 0.6 GA, Figure 2). The relative and absolute nadir of MAP at the end of occlusion was 29.5 ± 1.7% and 11.2 ± 0.7 mmHg (N.S. between groups).

**Figure 3:**
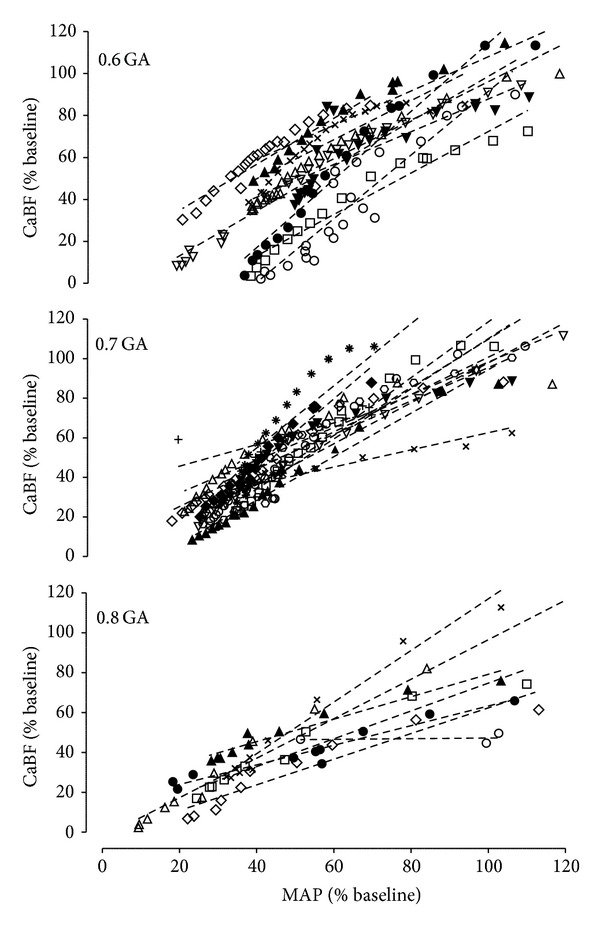
The relationship between changes in mean arterial pressure (MAP, % baseline) and carotid blood flow (CaBF, % baseline) from the minute MAP fell below baseline until the end of occlusion in 0.6 gestation (0.6 GA), 0.7 gestation (0.7 GA), and 0.8 gestation (0.8 GA) fetuses. Different symbols represent individual animals, while the dotted lines indicate their regression relationship. All fetuses showed a positive within-subjects relationship between mean arterial pressure and cephalic perfusion during the development of hypotension (*P* < 0.01, *R* ^2^ = 0.91, *n* = 28). The predictor variable gestational age was not significant.

Occlusion was associated with a rapid but brief rise in CaBF followed by a temporary fall before recovering back to baseline values at all gestational ages. CaBF fell progressively below baseline values from 5.9 ± 0.7 min onwards (N.S. between groups). This pattern closely paralleled changes in MAP. The rate of fall in CaBF during occlusion increased considerably with advancing gestation, such that 0.8 GA fetuses developed a significantly lower CaBF compared with the 0.6 GA and 0.7 GA groups from 6 and 7 min onwards, respectively (*P* < 0.05 versus 0.6 GA and 0.7 GA, Figure 2). There was a significant within-subjects correlation in all fetuses between the fall in MAP and the fall in CaBF (*P* < 0.01, *R* ^2^ = 0.91, *n* = 28, Figure 3), from the minute MAP fell below baseline values until the end of occlusion. This relationship was not significantly affected by gestational age. The relative nadir of CaBF at the end of occlusion was 22.1 ± 2.5% (N.S. between groups), while the absolute nadir was 2.4 ± 0.7 mL/min (0.6 GA), 9.7 ± 2.1 mL/min (0.7 GA), and 12.4 ± 3.2 mL/min (0.8 GA), respectively (*P* < 0.05, 0.6 GA versus 0.7 GA and 0.8 GA).

### 3.3. T/QRS Ratio and ST Waveform Analysis

**Figure 4:**
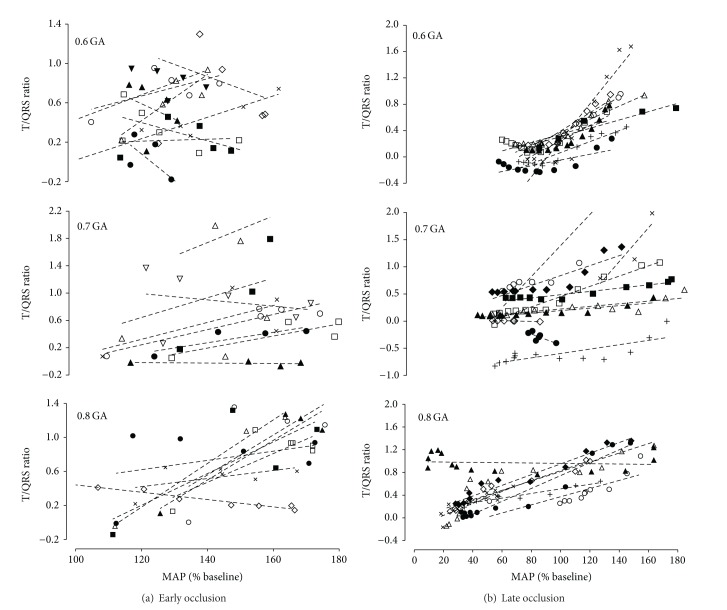
The relationship between changes in mean arterial pressure (MAP, % baseline) and T/QRS ratio in 0.6 gestation (0.6 GA), 0.7 gestation (0.7 GA), and 0.8 gestation (0.8 GA) fetuses. (a) shows the within-subjects relationship changes from the start of occlusion until the peak T/QRS ratio; the period when most important fetal cardiovascular adaptations take place (0.6 GA: N.S., *n* = 9; 0.7 GA: N.S., *n* = 8; 0.8 GA: *P* < 0.05, *R* ^2^ = 0.38, *n* = 8). (b) shows the within-subjects relationship changes from the peak T/QRS ratio until 15 minutes of occlusion; the period during which most of the fetal decompensation occurs (*P* < 0.01, *R* ^2^ = 0.67, *n* = 27). There was no significant effect of gestational age. Different symbols represent individual animals, while the dotted lines indicate the within-subject regression relationship. Seven of 25 fetuses did not have a positive relationship between MAP and T/QRS ratio during the early phase of occlusion.

All fetuses showed a rapid elevation of T/QRS ratio at the onset of occlusion, reaching a peak of 1.1 ± 0.2 at 3.7 ± 0.3 min (N.S. between groups). This rise was more rapid at 0.8 GA, such that the T/QRS ratio was significantly higher compared with 0.6 GA during the first and second minute (*P* < 0.05, Figure 2) and greater than 0.7 GA during the first minute of occlusion (*P* < 0.05, Figure 2). There was a significant within-subject correlation between the rise in T/QRS ratio and increase in MAP at 0.8 GA, from the start of occlusion until the peak increase of T/QRS ratio (0.6 GA, N.S., *R* ^2^ = 0.19, *n* = 9; 0.7 GA, N.S., *R* ^2^ = 0.22, *n* = 8; 0.8 GA, *P* < 0.05, *R* ^2^ = 0.38, *n* = 8, Figure 4). As occlusion continued, the T/QRS ratio fell gradually at all gestational ages and in nearly all cases had resolved to baseline values by the end of occlusion. This fall was markedly slower at 0.8 GA, so that the T/QRS ratio was greater than 0.6 GA between 9 and 11 min (*P* < 0.05, Figure 2) and greater than 0.7 GA between 8 and 10 min (*P* < 0.05, Figure 2). There was a significant within-subjects correlation between this gradual resolution of T/QRS ratio and the fall in MAP, from the peak T/QRS ratio until 15 minutes of occlusion (*P* < 0.01, *R* ^2^ = 0.67, *n* = 27, Figure 4), with no significant effect of gestational age.

Five of 10 fetuses in each of the younger groups and all animals in the 0.8 GA group showed periods of T wave inversion during the baseline period. None of these fetuses had their occlusion terminated early. Four (0.6 GA) and 3 (0.8 GA) fetuses responded to the onset of occlusion with a pronounced but brief period of marked T wave inversion. Only one of these fetuses had not shown prior T wave inversion during the baseline period. All fetuses rapidly developed upright T wave elevation early during the occlusion. T wave height then gradually fell as occlusion continued, and the T wave became inverted again in 14 fetuses (4 fetuses at 0.6 GA, 6 fetuses at 0.7 GA, and 4 fetuses at 0.8 GA). Of these, twelve (3 fetuses at 0.6 GA, 5 fetuses at 0.7 GA, and 4 fetuses at 0.8 GA) maintained this inverted T wave profile during recovery. All fetuses that had their occlusion released early and all fetuses that received adrenalin at 0.8 GA for cardiac resuscitation showed prolonged periods of T wave inversion during the recovery period. Other ST waveform shape changes such as biphasic waveforms or ST depression were not observed in any fetuses at any time before, during, or after the occlusion.

## 4. Discussion

The present study confirms that umbilical cord occlusion resulted in a rapid increase in T/QRS ratio that coincided with initial hypertension and bradycardia at all gestational ages. This increase in T/QRS ratio was significantly faster at 0.8 gestation and associated with relative greater increase in blood pressure at this age. The initial hypertension at the beginning of occlusion was associated with a brief increase in CaBF followed by maintenance around baseline values until approximately 6 minutes at all gestations. With ongoing occlusion, FHR, MAP, and CaBF fell progressively and the T/QRS ratio began to fall towards baseline values.

Severe hypotension and cephalic hypoperfusion during occlusion developed more rapidly towards term gestation than in immature fetuses, consistent with our previous findings [13]. From 8 minutes of occlusion onwards, the fall in MAP below baseline was strongly correlated with a concordant fall in CaBF at all gestational ages. The gradual fall in T/QRS ratio was much slower at 0.8 gestation, so that the T/QRS ratio was higher than in the younger groups after MAP fell below baseline values.

There was a strong within-subjects correlation between the early rise in T/QRS ratio and increase in MAP at 0.8 gestation, and overall, between the subsequent resolution of the T/QRS elevation and the progressive fall in MAP in the great majority of fetuses; this relationship was not affected by gestational age. Taken together, these findings suggest that the T/QRS ratio is a reliable index of fetal hypoxic stress early on during severe asphyxia at 0.8 gestation, although it was a poor predictor of subsequent failure of fetal adaptation.

Severe acute insults, such as abruptio placentae and umbilical cord prolapse, are significant causes of perinatal morbidity [38]. Conceptually, there are two distinct phases to the cardiovascular responses of the fetus to acute asphyxia: an immediate chemoreflex response that triggers vagally mediated bradycardia and peripheral vasoconstriction through alpha-adrenergic efferents [39], which results in early hypertension that is further augmented by the release of catecholamines from the adrenal medulla [40]. This is followed by a longer period of progressive hypoxic decompensation that is ultimately terminated by profound hypotension [39, 41, 42]. The chemoreflex response, which helps to reduce myocardial workload through centralization of blood flow to the heart, brain, and adrenals [43], has been described in fetal sheep from very early in gestation [44] and does not differ greatly throughout late gestation during severe asphyxia [13].

We now demonstrate that the marked initial hypertension during umbilical cord occlusion corresponded with only a transient increase in cephalic blood flow at all gestations and was followed by maintenance at around baseline values from 2 to 7 minutes of occlusion. Other studies have demonstrated a similar cerebrovascular response to umbilical cord occlusion and common uterine artery compression (flow reductions below 25% of baseline) in the mature fetal sheep, with either no increase or a moderate fall in carotid blood flow [5, 45, 46], despite initial hypertension. Speculatively, this actively-mediated restriction of blood flow may help prevent acute cerebral hemorrhage from cephalic hyperperfusion [9]. The brief period of cephalic hyperperfusion in the present study had resolved by two minutes of occlusion, suggesting that it was primarily related to the initial hypertension, before carotid vascular resistance became maximal.

Perhaps more surprising was that CaBF fell below its relative baseline two minutes before MAP reached baseline. This phenomenon was seen in all gestational ages and coincided with gradual peripheral vasodilatation [13]. Nevertheless, the relative decline in MAP was closely associated with a corresponding fall in CaBF from the minute blood pressure dropped below baseline values and was ultimately terminated by severe hypotension and cephalic hypoperfusion at the end of occlusion. This was not different across gestations. Our observations are consistent with previous evidence in fetal sheep that suggest that the fall in cerebral perfusion during asphyxia reflects loss of cerebral autoregulation [47, 48]. For example, Szymonowicz et al. reported that resting blood pressure (37 mmHg) was very close to the lower end of the autoregulatory range in the immature fetal sheep [8]. Even in 118–122 day old healthy fetal sheep the relationship between cerebral blood flow and arterial pressure is reported to be linear between 18 and 45 mmHg, and normal, resting blood pressure was very close to the lower end of the autoregulatory range [11]. Further, hypercarbia can abolish regional cerebral autoregulation in healthy piglets [12]. Taken together, these findings highlight the vulnerability of the developing fetus to hypoperfusion when hypotension develops during asphyxia.

A potential limitation of this study is that carotid blood flow was used as an indirect index of cephalic blood flow. Carotid blood flow has been shown to correlate well with microsphere and laser Doppler measurements during physiological manipulations [30, 31, 49]. Nevertheless, a substantial proportion of carotid artery blood flow goes to nonbrain tissues [31]. Thus, potentially, a larger fraction of carotid flow perfusing nonbrain tissues such as the face and scalp might have been diverted to the brain during asphyxia than was evident from carotid blood flow measurements. Such an effect could mask an underlying real increase in cerebral flow.

Importantly, the present study shows that initial cardiovascular adaptation coincides with an early rise in T/QRS ratio, while progressive systemic decompensation is accompanied with a gradual resolve of T/QRS ratio. This is consistent with previous studies in fetal sheep at 91–94 and 123–129 days that showed a brisk transient increase in T/QRS ratio associated with initial hypertension during asphyxia, followed by reduction in T/QRS ratio as hypotension developed and metabolic acidosis worsened [17, 20]. Elevation of the ST segment in the electrocardiogram (measured relative to the QRS complex and expressed as the T/QRS ratio) has been suggested as a useful diagnostic marker for asphyxia in both experimental and clinical monitoring studies [17, 20, 50–52]. Mechanistically, the area between the ST segment and T wave corresponds to the transition from ventricular depolarization to repolarization and is an energetically demanding process. The relationship between asphyxia and ST segment changes is only partially understood, but it likely reflects altered ionic cellular currents resulting from anaerobic myocardial glycogenolysis, partially mediated via *β*-adrenoreceptor stimulation [15, 18, 24, 25]. In acutely exteriorized fetal lambs at least, the rate of myocardial glycogenolysis during graded hypoxia, with depletion of glycogen and ATP, correlated directly with the degree and rate of ST segment elevation [18, 19].

In the present study, the immediate rise in T/QRS ratio was significantly greater at 0.8 GA during the first few minutes of occlusion compared with the younger groups and associated with greater hypertension at a time when peripheral vasoconstriction was almost maximal [13]. These data are in agreement with evidence that levels of circulating catecholamines are markedly higher in mature fetuses during hypoxia and are correlated with T/QRS height [24, 53]. Presumably, greater catecholamine levels would favor anaerobic myocardial glycogenolysis to meet higher metabolic demand in the near term fetuses [18]. Nevertheless, the absence of a direct relationship between MAP and T/QRS ratio early during asphyxia in the present study demonstrates that although ST segment elevation broadly coincides with initial fetal adaptation, it is not significantly linked to hypertension in the immature fetus. Even at 0.8 gestation this correlation was relatively weak.

The exact significance of the progressive decline in ST segment with continuing umbilical cord occlusion remains unclear. Intriguingly, to the best of our knowledge this phenomenon has only been reported during prolonged severe asphyxia [17, 20]. In mature lamb fetuses, brief maximal augmentation of the ST segment followed by a fall in amplitude was seen during severe hypoxia that was associated with fetal hypotension and reduced cardiovascular output; in that study the loss of ST height was closely correlated with severity of metabolic acidosis [19]. A limitation of the present study is that relative comparisons of severity of metabolic acidosis across gestations were not possible as blood gases were taken at different time points during the occlusions. Speculatively, a gradual metabolic depletion of glycogen during asphyxia could possibly lead to a progressive impairment in anaerobic cardiac metabolism [18]. This would not explain the more rapid resolution of T/QRS elevation in immature fetuses in the present study, since cardiac glycogen stores are much greater at midgestation [22, 23]. Further, Cheung reported a near linear increase in fetal adrenaline and noradrenaline levels during 30 minutes of maternal inhalational hypoxia, and thus it seems improbable that there could have been a fall in catecholamines during prolonged asphyxia [54].

We observed a slower fall in T/QRS ratios at 0.8 gestation during the hypoxic decompensation phase of prolonged severe asphyxia, when hypotension was significantly more severe than in the younger groups. The capacity of the immature fetus to maintain systemic pressure more effectively during asphyxia has been partially attributed to greater anaerobic capacity and lower basal metabolic activity [21, 22]. Thus the greater T/QRS ratio at 0.8 gestation in this critical phase may reflect relatively greater anaerobic myocardial activity, with accelerated consumption of glycogen reserves. MAP is a function of combined ventricular output and vascular resistance, and combined ventricular output is a product of fetal heart rate and stroke volume. Since, peripheral vascular resistance in this phase was greater in near-term compared to immature fetuses [13] and the relative and absolute FHR were highly similar between gestations, this denotes that greater hypotension in 0.8 GA fetuses is presumptively due to greater impairment of contractility leading to reduced stroke volume [55]. We propose that at 0.8 gestation fetuses required greater anaerobic myocardial metabolism to maintain cardiovascular output, resulting in a greater rise in T/QRS ratio.

Although the gradual decrease in T/QRS ratio was correlated with the fall in MAP during occlusion, this did not clearly correspond with the onset of hypotension at any gestational age in this study. Thus, this was not a strong index of loss of fetal adaptation. Further, we did not observe biphasic waveforms or ST segment depression, although these have been suggested to be associated with cardiac compromise [26]. The loss of ST segment elevation and appearance of inverted T waves during umbilical cord occlusion and graded hypoxia has been previously interpreted as markers of reduced myocardial contractility and serious cardiac dysfunction [20, 28]. Although we observed inverted T waves during recovery in all the fetuses that had occlusion released early, and all 0.8 GA fetuses who were given adrenalin for cardiac resuscitation, 12 fetuses that showed T wave inversion recovered from occlusion despite severe hypotension. Thus, although this feature most likely represented reduced myocardial contractility, it was not unique to fetuses that developed severe myocardial compromise nor did it identify those fetuses with reduced myocardial contractility alone.

In conclusion, prolonged asphyxia resulted in progressive metabolic acidosis and severe hypotension; the rate of development of hypotension and associated cephalic hypoperfusion increased toward term gestation. This was further associated with greater T/QRS ratio during early adaptation and late hypoxic decompensation. Better cardiovascular adaptation during asphyxia is associated with improved myocardial anaerobic metabolism and cephalic perfusion. An increase in T/QRS ratio is a reasonable marker of asphyxia during early adaptation at 0.8 gestation, but it does not seem to be an effective marker of developing fetal hypotension and cerebral hypoperfusion.

## Figures and Tables

**Table 1:** Baseline physiological data.

Group	Weight (g)	T/QRS ratio	FHR (bpm)	MAP (mmHg)	CaBF (mL/min)
0.6 GA	1283 ± 155^∗†^	0.03 ± 0.0^†^	189.5 ± 3.4^†^	35.5 ± 0.7^∗†^	11.0 ± 1.5^∗†^
0.7 GA	1623 ± 126^‡^	0.04 ± 0.0^‡^	191.3 ± 4.0^‡^	37.3 ± 0.5^‡^	42.2 ± 3.8^‡^
0.8 GA	3329 ± 170	−0.11 ± 0.0	171.7 ± 5.5	43.6 ± 0.6	59.1 ± 7.5

GA: gestation; FHR: fetal heart rate; MAP: mean arterial pressure; CaBF: carotid blood flow. Baseline values represent averaged data of one hour before occlusion. Data are means ± SEM; between group comparisons by one-way ANOVA and LSD test. *P < 0.05; 0.6 GA versus 0.7 GA, ^†^ *P* < 0.05; 0.6 GA versus 0.8 GA, and ^‡^ *P* < 0.05; 0.7 GA versus 0.8 GA.

**Table 2:** Blood composition parameters.

	Group	Baseline	Occlusion time 1	Occlusion time 2	Recovery
pH	0.6 GA	7.39 ± 0.0	7.06 ± 0.0^†^	6.76 ± 0.0^†^	7.36 ± 0.0*
	0.7 GA	7.38 ± 0.0	7.05 ± 0.0^†^	6.82 ± 0.0^†^	7.31 ± 0.0^†^
	0.8 GA	7.36 ± 0.0	7.21 ± 0.0^†^	6.94 ± 0.0^†^	7.18 ± 0.1^†^

PaO_2_ mmHg	0.6 GA	25.1 ± 0.6	7.1 ± 1.0^†^	8.4 ± 0.9^†^	26.1 ± 0.9
	0.7 GA	22.3 ± 0.9	6.0 ± 0.7^†^	7.0 ± 0.6^†^	29.6 ± 2.4^†^
	0.8 GA	19.6 ± 1.6	5.4 ± 0.9^†^	8.3 ± 0.9^†^	23.0 ± 3.2

PaCO_2_ mmHg	0.6 GA	44.5 ± 0.8	90.5 ± 3.8^†^	159.5 ± 3.3^†^	42.8 ± 0.8
	0.7 GA	47.7 ± 1.3	96.6 ± 3.6^†^	150.8 ± 7.1^†^	43.4 ± 1.0
	0.8 GA	45.7 ± 2.4	71.1 ± 4.0^†^	114.7 ± 2.6^†^	49.9 ± 1.2

Lactate mmol/L	0.6 GA	1.0 ± 0.3	4.5 ± 0.4^†^	7.4 ± 0.5^†^	3.0 ± 0.3^†^
	0.7 GA	0.7 ± 0.0	3.8 ± 0.2^†^	7.3 ± 0.2^†^	4.9 ± 0.3^†^
	0.8 GA	1.0 ± 0.3	2.5 ± 0.4	5.5 ± 0.2^†^	6.4 ± 1.6^†^

Glucose mmol/L	0.6 GA	1.1 ± 0.1	0.4 ± 0.1^†^	0.4 ± 0.1^†^	1.3 ± 0.1
	0.7 GA	0.9 ± 0.0	0.3 ± 0.0^†^	0.6 ± 0.1^†^	1.6 ± 0.1^†^
	0.8 GA	0.7 ± 0.1	0.3 ± 0.1	1.0 ± 0.1	1.5 ± 0.3^†^

Blood samples were taken 60 min before asphyxia (baseline), during the occlusion period (0.6 GA; 5 and 25 min, 0.7 GA; 5 and 20 min, 0.8 GA; 2 and 12 min), and 60 minutes after asphyxia (recovery). PaO_2_, fetal arterial pressure of oxygen; PaCO_2_, fetal arterial pressure of carbon dioxide. Data are means ± SEM; comparisons to baseline by one-way ANOVA and Dunnett test. *P < 0.05 versus baseline; ^†^ *P* < 0.01 versus baseline.
